# Development of a Synthetic 3D Platform for Compartmentalized Kidney In Vitro Disease Modeling

**DOI:** 10.1002/adhm.202503287

**Published:** 2025-10-23

**Authors:** Ninon Möhl, Daphne Bouwens, Johanna Abele, Aline Hans, Tanja Topic, Daniel Günther, Jitske Jansen, Rafael Kramann, Laura De Laporte

**Affiliations:** ^1^ Institute for Technical and Macromolecular Chemistry, RWTH Aachen University Chair for Macromolecular Materials for Medicine 52074 Aachen Germany; ^2^ DWI–Leibniz Institute for Interactive Materials 52074 Aachen Germany; ^3^ Department of Medicine 2 (Nephrology, Rheumatology, Clinical Immunology, Hypertension) RWTH Aachen University Faculty 52074 Aachen Germany; ^4^ Department of Internal Medicine, Nephrology, Transplantation Erasmus Medical Center 3015 Rotterdam Netherlands; ^5^ Institute of Applied Medical Engineering, Department of Advanced Materials for Biomedicine RWTH Aachen University Hospital 52074 Aachen Germany

**Keywords:** 3D cell culture, in vitro disease modeling, microfluidics

## Abstract

3D in vitro tissue and disease models have emerged as an important tool for diagnostic and therapeutic screenings, as they offer a closer approximation toward native environments than traditional 2D cell culture. Kidney disease modeling in particular has progressed to using induced pluripotent stem cells (iPSCs) and microfluidic platforms to replicate the complex microenvironment of the kidney. However, current models lack mature tissue development, scalability, tunability, and spatial organization. In this study, a fully synthetic, 3D kidney disease platform that addresses these challenges is presented. This model comprises a compartmentalized poly (ethylene glycol) (PEG)‐based hydrogel matrix with anisotropic PEG‐based microgels. This multiphasic hydrogel system provides control over spatially organizing a triple‐co‐culture of key renal cell types: tubule‐epithelial cells (CD10^+^), endothelial cells (CD31^+^), and fibroblasts (PDGFRβ^+^). Structural control and compartmentalization are enabled through enzymatically degradable rod microgels produced using microfluidics, allowing for a modular system. This study characterizes the synthetic models and analyzes the functionality of the system by examining cell‐material interactions. The use of this system as a promising disease model is demonstrated through the addition of TGFβ, inducing fibrosis. This work highlights a novel approach to building a fully synthetic, scalable, modular kidney model with a tunable microenvironment.

## Graphical abstract

A fully synthetic, compartmentalized 3D kidney disease model is introduced. The kidney model combines a PEG‐based hydrogel matrix with anisotropic, enzymatically degradable rod‐shaped microgels to spatially arrange a triple co‐culture of key renal epithelial, endothelial, and fibroblast cells. Control over the microenvironment is given by the tunability of the platform. Proof of concept is presented via TGFβ‐induced fibrosis.

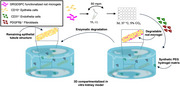

## 1 Introduction

3D in vitro disease models are becoming an important tool in tissue engineering and biomedical research as they provide a more accurate representation of the native tissue than conventional 2D monolayer cell cultures.^[^ ^1^ ^]^ These advanced in vitro models could allow for therapeutic screenings in healthy and diseased conditions on a large scale. Recent developments have expanded the potential of 3D in vitro models for multiple diseases, such as cancer, cystic fibrosis, cardiovascular diseases, and kidney diseases.^[^ ^2^, ^3^ ^]^ Kidney disease research in particular has made various advances modeling polycystic kidney disease (PKD), acute kidney injury (AKI), and fibrosis, using organoids from induced pluripotent stem cells (iPSC), as well as from adult stem cells.^[^ ^4^, ^5^, ^6^ ^]^

IPSC‐derived kidney organoids overcome multiple drawbacks from the conventional monolayer cell‐culture, as the iPSCs self‐differentiate into over 15 cell types of the kidney, and demonstrate complex cell‐cell and cell‐matrix interactions in 3D.^[^ ^7^, ^8^ ^]^ However, iPSC‐derived kidney organoids only embody human fetal kidney development,^[^ ^9^ ^]^ lack immune cells,^[^ ^4^ ^]^ and contain off‐target cell types because of uncontrollable iPSC differentiation.^[^ ^10^ ^]^ Recently, the potential of microfluidic‐based kidney models to stimulate maturation has also been studied,^[^ ^11^ ^]^ enabling the formation of perfusable channels. However, this strategy often relies on commercially available expensive chips with one‐time use and cannot be easily tuned to specific needs.^[^ ^11^, ^12^, ^13^ ^]^ Alternatively, other models have used bioprinters and a gelatin‐fibrin‐based extracellular matrix (ECM) to establish a 3D environment for tubule‐interstitium^[^ ^14^, ^15^ ^]^ or proximal tubule (PT) on perfusable chips.^[^ ^3^ ^]^ Kroll et al. recently further developed this system by adding a second channel for vascularization and using kidney organoids to better mimic the native tissue microenvironment.^[^ ^16^ ^]^

These biofabrication models, however, use animal‐ or plant‐derived materials, like GelMa^[^ ^17^ ^]^ or alginate.^[^ ^18^ ^]^ The properties of such materials are often difficult to control and can vary from batch‐to‐batch, which affects reliability and data reproducibility. As an alternative, a peptide‐based hydrogel has been developed to culture (hiPSC)‐derived kidney organoids,^[^ ^17^, ^19^ ^]^ representing a move toward more defined and tunable matrices. To the best of our knowledge, no fully synthetic polymer‐based 3D platform using a co‐culture of single cells has been established for in vitro kidney disease modeling. Developing such a model based entirely on synthetic materials offers clear advantages, including precise control over the microenvironment, modularity, lower costs, and easy integration with automated pipette systems for high‐throughput screening. Synthetic 3D hydrogels in particular exhibit high potential as they can easily be tuned (bio)chemically, and closely resemble the ECM due to their water‐swollen network.^[^ ^20^ ^]^ ECM‐mimicking materials and specific functional groups can be added as required, offering great control over gelation, mechanical, physical, and biochemical properties as well as its degradation mechanism and rate. Among synthetic materials, poly (ethylene glycol) (PEG) is one of the most common synthetic polymers for 3D cell culture inside hydrogels, as it is bioinert and biocompatible, and can be functionalized with defined bioactive molecules.^[^ ^21^ ^]^

Conventional hydrogels are intrinsically isotropic and do not offer any hierarchically structured organization, thus failing to recapitulate the complexities of the in vivo environment. Therefore, spatial guidance through structural compartmentalization is needed to generate efficient 3D functional physiological or pathological tissue models.^[^ ^22^ ^]^ For example, other reports have demonstrated that fibrosis is mechanistically depended on injured PT and spatial communication, during which the PT sends signals to the interstitial space, leading to activation of fibroblasts into myofibroblasts and fibrosis.^[^ ^23^, ^24^ ^]^ In order to spatially organize cells in a directed manner, our group has previously used cellulose nanofibrils (CNF) to create 3D scaffolds via extrusion sacrificial templating.^[^ ^25^ ^]^ Tubule CNF structures were coated with fibroblasts and degraded enzymatically using cellulase in a controlled manner at specific time points. Doing so, a 3D free‐standing cellular tube with a large lumen (≈0.8 mm diameter) could be formed, composed of fibroblasts. While the lumen size resulting from this method is too large for developing a new kidney model, the same principle can be applied using a sacrificial material with smaller dimensions. By embedding a small cell‐coated sacrificial material in a larger 3D hydrogel, 3D compartments featuring designed size, shape, and cell type can be created to better mimic the natural kidney environment.

In this work, we developed an entirely new synthetic compartmentalized 3D in vitro disease model and employed the kidney as our model system. To create a multiphasic anisotropic hydrogel system, we used plug flow microfluidics to produce sacrificial PEG‐based rod microgels that can be embedded inside a surrounding PEG‐based hydrogel matrix. This combination allows for a compartmentalized triple co‐culture, implementing three key renal cell types of tubule‐epithelial (CD10^+^) and endothelial (CD31^+^) cells, and fibroblasts (PDGFRβ^+^) to achieve the required cell‐cell interaction and tissue‐mimicking structures. The tubular epithelia compartments, mimicking kidney proximal tubuli, are provided by on‐demand enzymatic degradation of the rod microgels. We studied the material‐cell interaction to create the 3D compartmentalized kidney models and validated their feasibility as an in vitro system for fibrosis modeling.

## 2 Results

### 2.1 Rod Microgels Production Using Plug‐Flow Microfluidics

**Figure 1:**
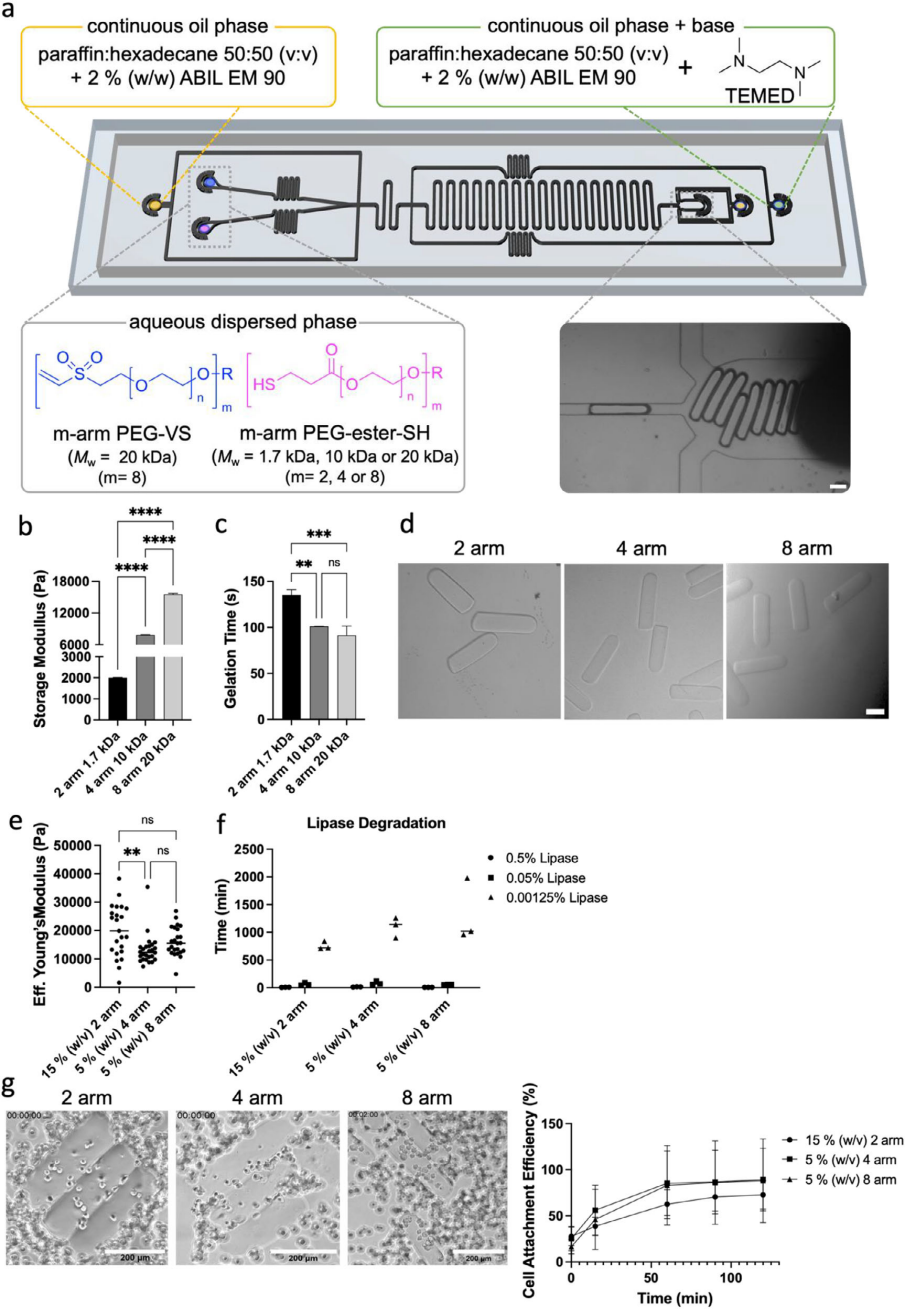
Microfluidic production of enzymatically degradable rod microgels and epithelial—microgel interaction. a) Plug‐flow microfluidic chip design,^[^ ^26^ ^]^ using Michael‐type addition. Different degradable pre‐polymers, such as linear and star‐PEG‐ester‐thiols (4 or 8‐arm), are combined with 8‐arm 20 kDa PEG‐VS to tune the microgel properties. Scale bar = 100 µm.  First, b) the storage modulus and c) gelation time of bulk hydrogels (5% (w/v) PEG) produced at pH 8 for the different precursor combinations were measured. e) Brightfield images of produced rod microgels with different m‐arm sPEG‐ester‐SH, scale bar = 100 µm, d) and their respective Young's moduli. f) Enzymatic degradation time of rod microgels using different lipase concentrations at 37 °C and 5% CO_2_ in EGM‐2 media. g) Cell attachment efficiency of CD10^+^ epithelial cells on GRGDSPC‐functionalized rod microgels with different compositions at 37 °C and 5% CO_2_ after 2 h, scale bar = 200 µm. Data are means ± standard errors. Statistical significance was determined by one‐way ANOVA with Tukey's multiple comparison test (^*^ *p* <0.05; ^**^ *p* <0.01; ^***^ *p* <0.001; ^****^ *p* <0.0001).

We developed a fully synthetic 3D compartmentalized kidney model, that enabled tubule‐like epithelial structures surrounded by a network of endothelial and fibroblast cells inside a 3D hydrogel. To emulate native kidney tubule structures, anisometric rod microgels were employed as structural templates that were obtained through plug flow microfluidics based on previous work from our group.^[^ ^26^ ^]^ The microfluidic design was refined by adding a focusing oil at the outlet of the channel to reduce pressure fluctuations upon gelation that influence the microgels’ properties (Figure **1**a).

The cross‐linking mechanism of the rod microgels is based on a Michael‐type thiol‐ene addition utilizing 8‐arm PEG‐vinylsulfone (PEG‐VS) and m‐arm star PEG‐ester‐thiol (m = 2, 4, or 8) (PEG‐ester‐SH), initiated through a basic pH using *N*,*N*,*N*,*N*‐tetramethylethylendiamin (TEMED). The introduction of an ester moiety to the network enabled hydrolytic degradation, as well as enzymatic degradation on demand using lipase.^[^ ^27^ ^]^ For all experiments, 8‐arm PEG‐VS (20 kDa) was used as a Michael‐acceptor, whereas the donor was altered with a different number of arms and molecular weight (Figure 1a). We selected different molecular weights (20, 10, and 1.7 kDa, for 8, 4, and 2‐arm, respectively) to keep the chain length constant. We wanted to understand how the polymer architecture (number of arms) affects the reactivity and performance in our microfluidic device. Prior to conducting microfluidic production, the bulk hydrogel properties of the precursors (5% (w/v) PEG) were evaluated through rheology. The storage modulus (G’) increased proportionally with the number of arms, with the highest G’ observed for 8‐arm PEG‐ester‐SH and the lowest for the linear PEG‐ester‐SH (Figure 1b). At the same time, the gelation time decreased (Figure 1c) with increasing functionality per molecule. These compositions were then applied to produce rod microgels (Figure 1d), where the total PEG concentration was now adapted to 15% (w/v) when using linear PEG‐ester‐SH, as below this concentration, no microgel production was possible. This is due to the long gelation time, which was observed for the bulk hydrogels. The microfluidic compositions, as well as the production parameters, can be found in Tables S1 and S2 (Supporting Information).

The produced rods are 80–120 µm in width and ≈300 µm in length (Figure S1, Supporting Information), which is more physiologically relevant than previously reported tubule‐like structures of >150 µm.^[^ ^16^, ^28^ ^]^ With microfluidics, diameters <100 µm can be achieved. The mechanical properties of the rod microgels were subsequently analyzed through nanoindentation (Figure 1e), where the composition with the linear precursor comprising the highest polymer concentration showed the highest Young's modulus, and the microgels made with 4 or 8‐arm PEG‐SH cross‐linker did not exhibit any significant difference. The latter is likely because the arm length of the employed stars remained the same (2.5 kDa). Interestingly, the swelling ratio did not correlate completely with the Young's modulus. Here, the linear samples swell the most, despite having the highest Young's modulus, and the 8‐arm samples swell the least (Figure S1, Supporting Information). This might be due to the arm length, as the linear samples exhibit shorter PEG chains and are thus more hydrophilic, enabling a higher incorporation of water.

### 2.2 CD10^+^ Cells are Unaffected by Lipase and Cover the Functionalized Microgels

As the rod microgels were employed to mimic kidney tubules and function as a template for kidney epithelium cells (CD10^+^), their on‐demand degradability was assessed through the addition of lipase, which is known to selectively degrade esters through ester hydrolysis.^[^ ^27^ ^]^ We exposed microgels to lipase in a range of concentrations of 0.00125 –0.5% (w/v) to observe their degradation time. We found that a higher enzyme concentration led to a faster degradation for all samples (Figure 1f); microgels were degraded in 10 min with a concentration of 0.5% (w/v), instead of 1 or 18 h with 0.05% or 0.00125% (w/v) lipase, respectively. The microgel composition did not show an influence on the degradation rate. The linear samples do not degrade more slowly than the star samples, despite the higher polymer concentration, which is probably linked to the network structure and high swelling degree.

We then assessed whether the CD10^+^ epithelia cells are compatible with different concentrations of lipase by performing a metabolic assay. No change in cell behavior or growth was observed, indicating that lipase does not have a direct effect on the CD10^+^ cells (Figure S2, Supporting Information).

To guarantee efficient cell attachment, rod microgels were post‐functionalized with a cell adhesive peptide GRGDSPC. Unfunctionalized rod microgels showed no cell attachment after three days (Figure S3, Supporting Information). Images for cell attachment were recorded for a period of 2 h (Figure 1g; Figure S4, Supporting Information), and quantification of the cell attachment efficiency indicated a higher efficiency for microgels made with 4 or 8‐arm PEG‐SH compared to the linear PEG cross‐linker. Based on these results, we chose the microgels produced with 8‐arm PEG‐SH for all subsequent cell experiments as they remained the thinnest, more closely mimicking the kidney tubuli.

### 2.3 Kidney Interstitium is Modeled in a Hydrogel, and CD10^+^ Cells Show Physiological Relevant Traits

As kidney epithelium is physiologically supported by interstitium, we next optimized the surrounding hydrogel matrix to culture kidney‐specific interstitium cells. The surrounding matrix consists of an enzymatically cross‐linked PEG‐based hydrogel. 8‐arm PEG‐VS (20 kDa) is functionalized with two peptides, Ac‐FKGGGPQGIWGQERCG‐NH_2_ = K‐peptide or NQEQVSPLERCG‐NH_2_ = Q‐peptide, that can covalently bind through activated Factor XIII (FXIIa).^[^ ^29^ ^]^

Inside this matrix, we chose a combination of renal endothelial and fibroblast cells to mimic the kidney interstitium. The interplay of epithelial cell injury with endothelial cells and fibroblasts is considered a driver of fibrosis and kidney functional decline.^[^ ^23^, ^24^ ^]^ To capture these aspects between three key renal cell types, we used immortalized and genetically tagged cell lines derived from human nephrectomies for our 3D co‐cultures. We previously demonstrated the origin and characteristics of each cell line, confirming the epithelial nature of the CD10^+^ cells, the endothelial nature of CD31^+^ cells, and PDGFRβ^+^ cells as precursors of myofibroblasts.^[^ ^15^ ^]^

**Figure 2:**
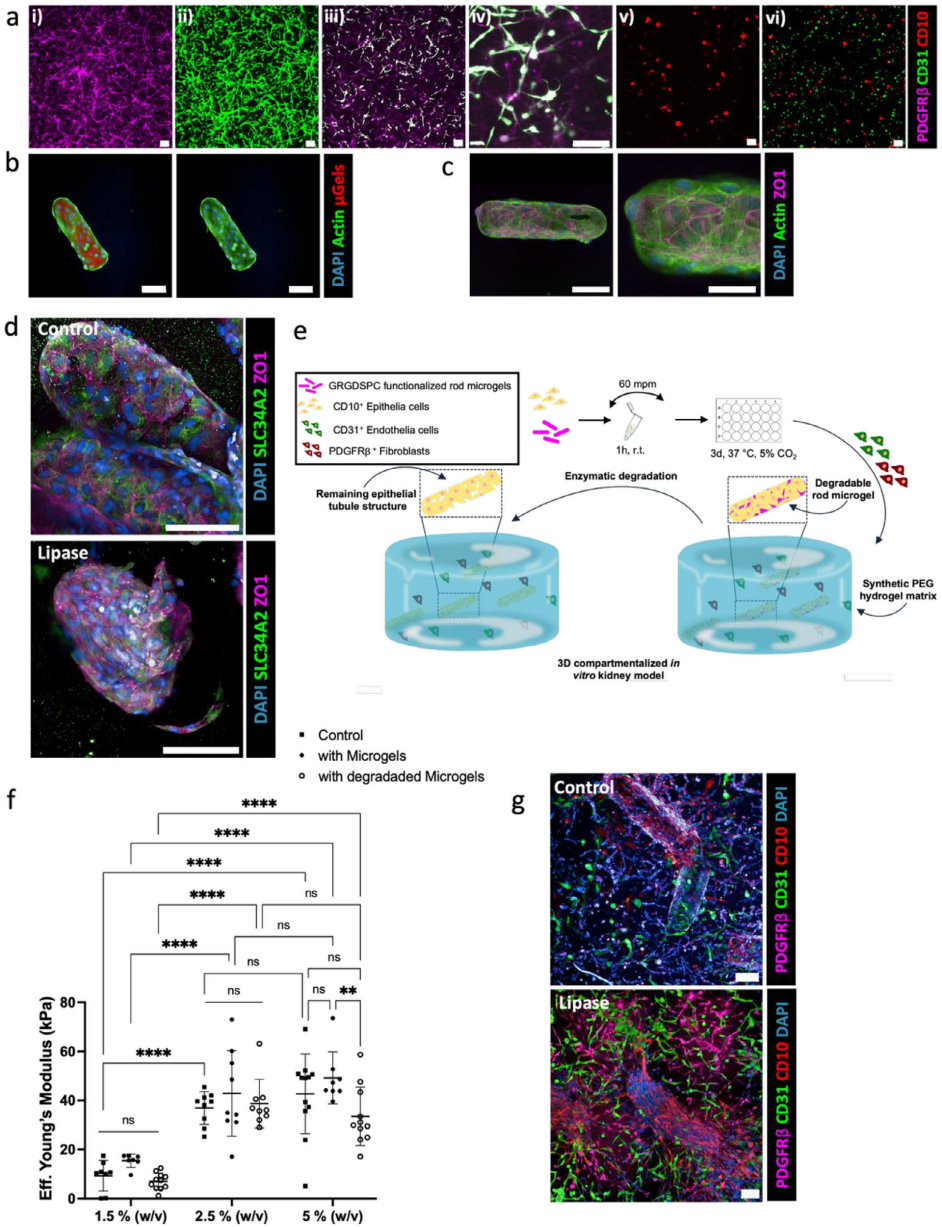
Cell—material interaction in 3D. a) Monocultures and co‐cultures of CD31^+^, PDGFRβ^+^, and CD10^+^ cells in a 1.5% (w/v) PEG‐QK hydrogel with 600 µm GRGDSPC and cultured for 7 days. i) mono‐culture of PDGFRβ^+^ or ii) CD31^+^ with 1000 cells µL^−1^, iii) co‐culture (CD31^+^+ PDGFRβ^+^) in a 1:1 ratio with 1000 cells µL^−1^ in total, iv) higher magnification of the cellular network formation, v) mono‐cultures of CD10^+^ with 1000 cells µL^−1^ in 3D without proliferation, vi) triple‐co‐culture (CD31^+^ + PDGFRβ^+^ + CD10^+^) (1:1:1, 500 cells µL^−1^ per cell type) inside the PEGQK hydrogel not exhibiting network formation. PDGFRβ^+^ signal was too weak to be displayed. Scale bars = 100 µm. b) Coating of CD10^+^ cells on GRGDSPC‐functionalized microgels after 3 days. Scale bars = 100 µm. c) ZO1 staining of CD10^+^ coated microgels to show epithelial junction formation after 3 days at two different magnifications. Scale bar = 100 µm (left), Scale bar = 50 µm (right). d) CD10+ coated microgels were transferred into a 1.5% (w/v) PEG‐QK matrix and cultured for 7 days. Lipase (0.000625% (w/v)) was added on day two and removed on day four to degrade the rod microgels. Microgels are not labeled. ZO1 and SLC34A2 staining show presence of epithelial tight junctions and NaPi‐IIb cotransporter in the control and after degradation. Scale bars = 100 µm. e) Procedure to produce 3D compartmentalized triple co‐culture hydrogel system. f) Effective Young's modulus of PEG‐QK hydrogels with different polymer concentrations and microgels before and after degradation through lipase. A control is provided without microgels. Data are means ± standard errors. Statistical significance was determined by two‐way ANOVA with Tukey's multiple comparison test (*p <0.05; **p <0.01; ***p <0.001; ****p <0.0001). g) Confocal images of 3D compartmentalized triple co‐culture system with PEG‐QK polymer concentrations of 1.5% (w/v) after 14 days of culture. Lipase (0.000625% (w/v)) was added on day two and removed on day five to degrade the rod microgels. Microgels are not labeled. Scale bars = 100 µm.

Culturing CD31^+^ and PDGFRβ^+^ cells as monocultures inside a 1.5% (w/v) PEG‐QK hydrogel for seven days exhibited network formation (Figure **2**a). For the co‐culture, different cell ratios were probed (1:1, 1:3, 1:2, 2:1, and 3:1; CD31^+^: PDGFRβ^+^ in a total of 1000 cells µL^−1^) (Figure S5, Supporting Information), and best network formation was observed at a 1:1 ratio (Figure 2a). The need for compartmentalization of the epithelial cells became clear when we observed no cell growth when CD10^+^ cells were added to the hydrogel as monoculture or as triple‐culture together with CD31^+^ and PDGFRβ^+^ cells, possibly due to signaling of the CD10^+^. As epithelium cells naturally form thin layers of tissue to line hollow structures inside the body, e.g. tubules, we aimed to better mimic this by creating a compartmentalized 3D hydrogel system, in which hollow structures would be pre‐formed using the degradable rod‐shaped microgels. For this, the GRGDSPC‐functionalized microgels were combined with CD10^+^ cells and cultured over three days, at which point the cells reached confluency on the microgel surfaces (Figure 2b). A ZO1 staining confirmed the presence of tight junctions that are responsible for sealing and preventing leakage of solutes and water (Figure 2c).

Next, to deepen biological validation and demonstrate the epithelial monolayer on the microgels, we transferred the CD10^+^ coated microgels into a 1.5% (w/v) PEG‐QK hydrogel matrix and assessed the presence of epithelial tight junctions and SLC34A2, a Na^+^/Pi cotransporter (NaPi‐IIb), before and after degradation of the rod microgels (Figure 2d). Similarly to microgels in suspension, we observed a confluent monolayer of CD10^+^ cells with tight junctions, and the presence of SLC34A2 indicates functional epithelial cells with their ability to take up phosphates with the cotransporter. To note, we lowered the lipase concentration from 0.00125% (w/v) to 0.000625%, as too rapid microgel degradation within 24 h (observed via the loss of the rhodamin B tag inside the microgel network) did not give the CD10^+^ cells enough time to attach to the surrounding hydrogel matrix and maintain their hollow tubular shape (Figure S6, Supporting Information).

In conclusion, the cell‐coated microgels were introduced into the hydrogel matrix system containing the CD31^+^ and PDGFRβ^+^ cells in a 1:1 ratio and cultured for 14 days. The microgels were enzymatically degraded at a chosen time during the culture using lipase to form the hollow structures (Figure 2e). We observed no effect of different lipase concentrations on the CD31^+^ and PDGFRβ^+^ cells using a metabolic assay (Figure S2, Supporting Information).

### 2.4 Stiffer Hydrogels Do Not Allow Proper Cell Growth and Network Formation

Hydrogel stiffness is a key factor of cellular behavior and structural organization. In our model, the stiffness might not only impact the CD31^+^ and PDGFRβ^+^ network formation, but also the CD10^+^ tubule‐like structure formation. Previous research has shown that renal tubule cells morphology differs depending on the substrate elasticity.^[^ ^30^ ^]^ To determine the influence of the surrounding hydrogel stiffness, we cultured our triple co‐culture in PEG‐QK polymer concentrations of 1.5, 2.5, or 5% (w/v). This resulted in hydrogels with increasing Eff. Young's moduli determined through nanoindentation of ≈9.29 ± 6.22, 36.92 ± 6.71, and 42.67 ± 16.23 kPa, respectively (Figure 2f). The larger standard deviations are inherent to this method as the gel was probed at different locations with 9 µm diameter colloidal probes. The addition of microgels to the hydrogels did not have a significant effect on the Eff. Young's modulus, also not after microgel degradation. Only for the stiffer 5% (w/v) hydrogels, the Young's modulus significantly reduces after microgel degradation. When forming the hydrogel with the CD10^+^‐coated microgels inside (day zero), lipase was added on day two (0.000625% (w/v)). After 14 days inside a 1.5% (w/v) PEG‐QK hydrogel, the triple co‐culture exhibits network formation, and the CD10^+^ cells keep the tubular shape given by the microgel (Figure 2g). For the stiffer surrounding hydrogels, we observed no cell growth or network formation of the CD31^+^ and PDGFRβ^+^ (Figures S7 and S8, Supporting Information).

Likewise, the CD10^+^ cells do not grow at higher PEG‐QK concentration than 1.5% (w/v). After microgel degradation, the CD10^+^ cell layers shrink and stretch over the void space, instead of keeping the rod shape of the microgel, resulting in a loss of the lumen. This result is in line with another study about kidney organoid lumen morphology inside an alginate‐based soft and dynamic hydrogel matrix, which showed stiffer hydrogel matrices (20 kPa) caused a loss of lumen structures, whereas softer matrices (0.5 kPa) supported the maturity of lumen structures and reduced mesenchymal cells.^[^ ^31^ ^]^ This finding shows that the elasticity of the hydrogel matters both in lumen formation and network formation, with softer substrate promoting both.

### 2.5 Primary Cilia of Epithelial Cells Transition From Outward to Inward after Degradation of the Microgel

**Figure 3:**
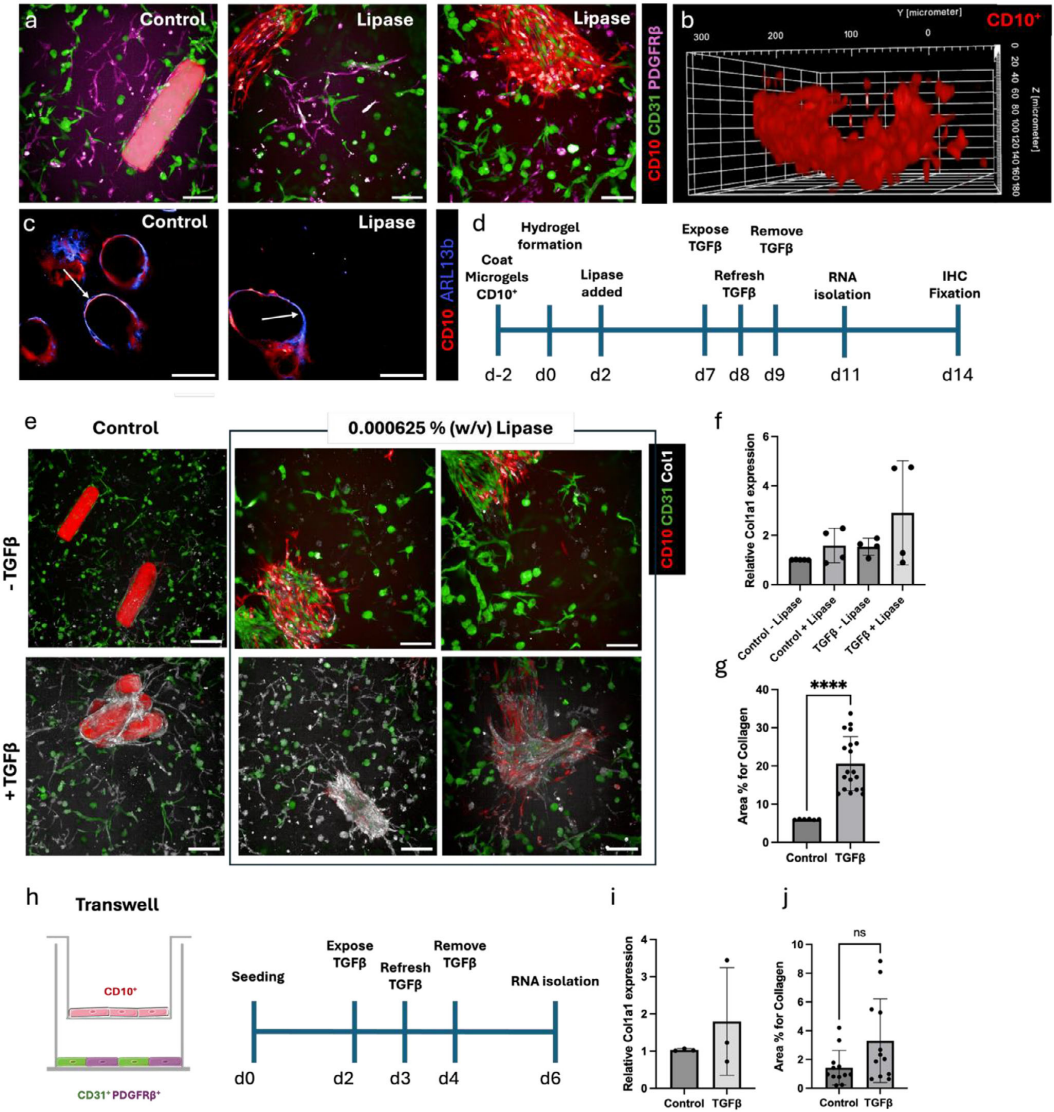
Injury stimuli 3D versus 2D. a) Anisotropic tubule‐like formation of CD10^+^ cells before degrading rod microgels (both CD10^+^ and microgel labeled red) (left). Degraded microgel inside compartmentalized triple co‐culture inside a 1.5% (w/v) PEG‐QK hydrogel (middle, right). Scale bar = 100 µm b) 3D view of the CD10^+^ remaining tubule‐like construct after degradation. c) Representative microscopy image of primary cilia (blue) before (control, unlabeled microgels) and after degradation (lipase) of the CD10^+^ (red) covered microgels. Arrows indicate orientation of cilia. d) Timeline of hydrogel formation and exposure to TGFβ. e) TGFβ‐treated and untreated 3D triple co‐cultures. Control with undegraded microgels (labeled) (left panels), CD10^+^ cells are depicted in red, CD31^+^ cells in green, Collagen1 in white, PDGFRβ not shown. Scale bars = 200 µm. f) Relative mRNA expression of Collagen1a1 in control and TGFβ‐exposed samples. Data are given as a mean and SD from 4 independent experiments each performed with 4 replicates. g) Collagen1 protein expression quantification in treated and untreated triple co‐culture in hydrogel after degradation of the microgels. Data depicted as percentage of area positive for Collagen1, ^****^ = *p* <0.0001, ns = p0.0566. h) Schematic overview of triple co‐culture in the transwell system and the experimental timeline. i) Relative mRNA expression of Collagen1a1 in control and TGFβ‐exposed samples in the transwell system. Data are given as a mean and SD from 3 independent experiments, each performed with 3 replicates. j) Collagen1 protein expression quantification in treated and untreated triple co‐culture in transwell system. Data depicted as percentage of area positive for Collagen1, ns = p0.0566.

Culturing the compartmentalized triple co‐culture (Figure **3**a) inside a 1.5% (w/v) PEG‐QK hydrogel showed that the CD10^+^ cells form a tubule‐like structure upon lipase‐induced microgel degradation (Figure 3a,b). The 3D view reveals lumen formation with a size of ≈100 µm; however, it should be noted that, at this time point, the structure is not fully enclosed by epithelial cells and remains porous at this stage. Next, we demonstrated the polarity of epithelial cells before and after degradation of the microgel. We found that prior to degradation, the basement membrane of the CD10^+^ cells is located toward the microgel surface, with the cilia pointing toward the surrounding hydrogel (Figure 3c). After degradation with lipase, the polarity is less clear. We propose that after degradation, the orientation of the cells is transitioning toward having the cilia on the apical side pointing toward the hollow lumen, in a manner consistent with physiological function.

### 2.6 TGFβ Exposure Leads To Fibrosis, Indicating the Potential for Kidney Injury Modeling

To assess the potential of our synthetic 3D in vitro system to model kidney disease, we stimulated the hydrogel network with TGFβ (Figure 3d). TGFβ is a known driver of fibrosis and has shown to initiate fibroblast‐to‐myofibroblast transition of the PDGFRβ^+^ cells in vitro.^[^ ^15^, ^32^ ^]^ After 48 h of TGFβ exposure, the hydrogel systems demonstrated an upregulation of the fibrosis readout Collagen1 both on a protein‐ and gene expression level (Figure 3e–g). These results also reveal that in control conditions, lipase did not affect collagen mRNA production (Figure 3f). Quantification of *Collagen1* relative mRNA expression revealed a ∼threefold increase in injured kidney models compared to the control (Figure 3f) after the microgels are degraded through lipase and TGFβ is added. Only a weak increase is observed by the addition of TGFβ when the microgels are not degraded. This is particularly interesting since it demonstrates the importance of lumen formation, which may create a more physiologically relevant interaction of tubule epithelial cells and fibroblasts in the initiation of fibrosis. Despite the mRNA levels being only slightly upregulated, Col1 protein quantification revealed a significant threefold increase in the injured kidney model with lipase compared to the control (Figure 3g).

To compare our engineered compartmentalized 3D microgel‐in‐hydrogel system to more conventional 2D co‐culture approaches, we used a 3D 6‐well transwell system with a triple co‐culture; using a 1:1 ratio of CD31^+^:PDGFRβ^+^ cells on the bottom, and CD10^+^ on the transwell (Figure 3h). After exposure of 48 h of TGFβ, we observed a twofold increase in relative *Col1a1* mRNA expression in the transwell system (Figure 3i), compared to a ∼threefold increase using our hydrogel model, indicating that the injury response may be enhanced in this model. We also assessed Col1 protein expression in the transwell system and observed a trend toward a modest increase of Col1 expression (*p* = 0.0566), although this was not of the same magnitude as in the hydrogels (Figure 3j; Figure S9, Supporting Information). Collectively, these results suggest the potential of our hydrogel model for drug development and future high‐throughput screening when using automated pipetting systems.

## 3 Discussion

In this study, we developed a fully synthetic compartmentalized PEG‐based hydrogel system including human‐derived kidney tubular epithelial structures surrounding degradable microgels, leading to representative hollow tubular structures. Our multiphasic hydrogel system is the first to provide precise control over spatially organizing key renal cell types and both the microgels and surrounding hydrogel support the specific cell growth. After degradation of the microgels to mimic the tubule interstitium, we demonstrated its potential in modeling kidney fibrosis and showed an increased response in *Collagen1* production in comparison to other 2D culture methods. Therefore, the model allows for a relevant cellular response to injury, suggesting that our system is valid and suitable for drug testing.

Previous studies have shown that (visco‐)elasticity and the 3D microenvironment matter for cell growth,^[^ ^33^, ^34^, ^35^ ^]^ which became apparent when probing softer versus stiffer hydrogel matrices. The softer hydrogel matrix promoted cell growth and network formation, as well as lumen formation, which is in line with previous findings when culturing kidney organoids inside alginate‐based hydrogels.^[^ ^31^, ^36^ ^]^ The stiffer substrates, on the other hand, did not exhibit cell spreading for both the endothelial and pericyte cells. Furthermore, the epithelial layer underwent shrinkage after microgel degradation inside the stiffer microgels, which was not the case for the softer hydrogels. Interestingly, even though we observed that the epithelial cells grow on the entire microgel surface before incorporation inside the surrounding hydrogel, upon degradation of the microgels, the epithelial cells do not result in a perfect lumen fully enclosing the space left by the degraded microgel. This could be due to insufficient attachment of the epithelial cells to the surrounding hydrogel matrix once they lose the microgel support. As we already know that the hydrogel stiffness influences the lumen formation, other drivers such as matrix viscoelasticity might play an important role, which will be studied in further studies. Currently, the hydrogel is completely elastic, lacking the viscoelasticity present in the native tissue. In literature stress‐relaxing hydrogels have shown to influence cilia frequency and length, as well as epithelial‐mesenchymal transition of kidney organoids.^[^ ^31^ ^]^

Kidney injury and fibrosis is characterized by a progressive accumulation of ECM proteins, leading to tubulointerstitial scarring and loss of renal function. The activation of human‐derived fibroblasts leading to an increased expression of the extracellular matrix component Collagen1 in our model shows that it provides a physiologically relevant 3D microenvironment that is responsive to fibrotic cues. The upregulation of Collagen1 observed in our system indicates active matrix remodeling and mimics early fibrotic events, making it a valuable platform for studying the progression of fibrosis and responsiveness to drugs. Utilizing only human‐derived kidney cells enhances the physiological relevance. Moreover, the tunability of the hydrogel stiffness and potential to introduce viscoelastic components enable future work to probe how mechanical cues contribute to fibrogenesis, which has been identified as a major driver in chronic kidney disease.

While our hydrogel model provides a new tunable and reproducible platform, it does not yet fully capture the complexity of in vivo kidney disease, particularly the influence of the immune cell populations or fluid flow, as incorporating immune cells into the culture media would more fully recapitulate the fibrotic niche. Furthermore, the addition of growth factors can support vascularization in 3D hydrogel systems^[^ ^37^ ^]^ and further improve the functionality of our platform. In addition, to better represent the size of tubules in vivo, the diameter of the microgels could potentially be reduced. Our system currently consists of ≈100 µm microgels covered in epithelial cells for tubule formation, while 25–40 µm is standard in human kidney architecture. Bioprinting such small open channels still remains a challenge, and to achieve smaller microgels, different microfluidic techniques would need to be employed, such as compartmentalized jet polymerization^[^ ^38^ ^]^ or in‐mold polymerization. However, so far, these methods have not used the chemistry employed in this report to enable the on‐demand enzymatic degradation to create hollow lumens.

In the future, our platform could potentially be used to model different types of kidney injury, including hypoxia for AKI or IL1β for inflammatory kidney injury. Lastly, as our model is fully synthetic, it could be a valuable platform for iPSCs culture^[^ ^39^ ^]^ to directly have spatial organization for more efficient differentiation and less off‐target cell populations, as this has been shown to support the growth of IPS‐derived intestinal organoids,^[^ ^40^ ^]^ and also affect kidney^[^ ^19^ ^]^ development. Moreover, this compartmentalized hydrogel system with tunable properties allows for a broadly applicable platform for drug testing in a reproducible and high‐throughput manner.

## 4 Experimental Section

### Chemicals and Cell Culture Material

ABIL EM 90 (Evonik Nutrition, Essen, Germany), 8‐arm poly (ethylene glycol) PEG‐OH 20 kDa (Creative PEGWorks, Chapel Hill, NC, USA, ≥95%), 4‐arm PEG‐OH 10 kDa (Creative PEGWorks, ≥95%), PEG‐OH 1.7 kDa (Sigma–Aldrich, St. Louis, MO, USA, ≥95%), 8‐arm PEG‐vinylsulfone 20 kDa (Creative PEGWorks, ≥95%), Sylgard 184 silicone elastomer kit (The Dow Chemical Company), Dulbecco's modified Eagle medium (DMEM, Gibco, Grand Island, NY, USA, EGM‐2 medium (Lonza, # EBM‐2 cc‐3154, EGM‐2 singleQuots Supplements cc‐4176), TGFβ (100‐21‐10UG, Peprotech), ethanol (VWR‐chemicals, Radnor, PA, USA, ≥98%), Novec 7500 (Sigma–Aldrich), paraffin (VWR‐chemicals), paraformaldehyde (PFA) (Sigma–Aldrich), phosphate buffer saline (PBS, pH 7.4, *c* = 1x, TGFβ (100‐21‐10UG, Peprotech), Thermo Fisher Scientific, Waltham, MA, USA), SYLGARD 185 silicone elastomer kit (Dow, Midland, MI, USA), methacryloxyethyl thiocarbamoyl rhodamine‐B (Polysciences, Warrington, PA, USA), *N*,*N*,*N*’,*N*’ – Tetramethylethylenediamine (TEMED, Sigma–Aldrich, ≥99%), *n*‐hexane (Sigma–Aldrich, ≥98%), *iso‐*propanol (Sigma–Aldrich, ≥98%), 3‐mercaptopropionic acid (Sigma–Aldrich, ≥99%), p‐toluenesulfonyl acid monohydrate (*p*‐TsOH) (Sigma–Aldrich, 98.5%), cyclohexane (Sigma–Aldrich, ≥98%), sodium bicarbonate (Sigma–Aldrich), GRGDS‐PC H–Gly–Arg–Gly–Asp–Ser–Pro–Cys–OH (trifluoroacetate salt) (CPC Scientific, Sunnyvale, CA, USA, ≥98%), Corning Costar Ultra‐Low Attachment Multiple Well Plate (Sigma–Aldrich), Protein LoBind Tubes (Eppendorf, Hamburg, Germany), Tridecafluoro‐1,1,2,2‐tetrahydrooctyltrichlorosilane (Sigma–Aldrich, 97%), acetone (Sigma–Aldrich, ≥98%), dichloromethane, ether (Sigma–Aldrich), dimethyl sulfoxide (DMSO, Sigma–Aldrich, ≥98%), polytetrafluoroethylene (PTFE) tubing (Instech), Lipase B Candida antarctica, recombinant from Aspergillus, 9.7 U mg^−1^ (SKU: 62288‐50mg‐F, Sigma–Aldrich)

### Synthesis of m‐Arm Poly (Ethylene Glycol) Ester Thiol

m‐arm PEG‐ester‐SH (m = 2, 4 or 8) were prepared by Fischer esterification of the respective PEG‐(OH)_m_ according to the literature.^[^ ^41^ ^]^ Briefly, in a 250 mL two‐neck round‐bottom equipped with a Dean‐Stark PEG‐(OH)_m_ (1500 linear difunctional PEG‐OH = 2‐arm or 10 000 Da sPEG‐OH 4‐arm or 20 000 Da sPEG‐OH 8‐arm) (1 eq., 1.00 g) was dissolved together with 3‐mercaptopropionic acid (40 eq.) and *p*‐toluenesulfonic acid *(p*‐TsOH) (0.1 eq.) in 70 mL cyclohexane. The reaction was refluxed at 130 °C for 24 h, and at least two volumes of Dean‐Stark were removed. Afterward, the reaction mixture was cooled down to r.t. and the cyclohexane was separated from the product via decantation. The remaining white wax was dissolved in DCM (50 mL) and washed with saturated NaHCO_3_ (2 x 15 mL), brine (1 x 15 mL), and dried over MgSO_4_. The solid was removed by filtration, and the solution was concentrated until a viscous oil was obtained. The resulting oil was dissolved again in minimal amount of DCM (2–3 mL) and precipitated by dropwise addition in excess cold (−20 ◦C) diethyl ether (40x vol DCM), and the final product was isolated as a white powder (isolated yield 70%). 1H NMR (CDCl3, 400 MHz): δ (ppm) = 4.26 (4H, m, ─CH2CH2OC(O)─), 3.64 (133H, br), (CH2CH2O)n), 2.77 (4H, m, ─CH2CH2SH), 2.68 (4H, m, ─CH2CH2SH), 1.68 (2H, t, SH).

### Rheological Measurements of Bulk Hydrogels

Rheological measurements of bulk hydrogels were performed using an HR‐3 rheometer PHR3 (TA Instruments, New Castle, DE, USA) at room temperature using a 20 mm conical 2° geometry probe. 8‐arm PEG‐VS 20 kDa and m‐arm PEG‐ester‐SH were mixed in phosphate buffer (100 mM, pH 8) and pipetted onto the device. Time sweeps were conducted at 1 Hz and 1% strain until a plateau was reached. Subsequently, frequency sweeps at 1% and strain sweeps at 1 Hz were performed.

### Microfluidic Device Preparation

As described elsewhere,^[^ ^26^ ^]^ microfluidic masters were produced via soft lithography. The polydimethylsiloxane (PDMS) mold was prepared using the Sylgard 184 silicone elastomer kit in a 10:1 ratio with the curing agent. The components were mixed and degassed using a PDMS mixer (Thinky PDMS Mixer, Thinky USA Inc., Laguna Hills, CA, USA) (mixing: 2000 rpm for 2 min; defoaming: 2200 rpm for 2 min). The mixture was subsequently poured onto the microfluidic silicon master and degassed by placing it in a desiccator under vacuum (10^−3^ bar). The PDMS was cured at 60 °C for 12 h. After curing, the PDMS was cut out, and holes for inlets and outlets of the channels were punched (biopsy puncher, inner diameter 0.75 mm, Sigma–Aldrich). The PDMS chip was washed three times with *iso*propanol and water. A glass slide (75 x 50 x 0.16 mm, Carl Roth, Karlsruhe, Germany) was washed three times with *iso*propanol and acetone. The PDMS mold and glass slide were treated with oxygen plasma to covalently bind both parts inside an oxygen plasma oven (PVA TePla 100E, PVA TePla, Wettenberg, Germany) at an oxygen flow of 28 mL mi^−1^n for 40 s at 100 W and 0.2 mBar. The completion of the bonding was done in an oven at 60 °C for 3 h. After finalization of the bonding, the microfluidic channels were rendered hydrophobic by chemically depositing 50 µL of tridecafluoro‐*1*,*1*,*2*,*2*‐tetrahydrooctyltrichlorosilane inside a desiccator at 10^−3^ bar overnight. The remaining residues of the silane were removed using paraffin.

### Preparation of Microfluidic Solutions

The oil phase consisted of 50:50 (v/v) hexadecane/paraffine and 2% (w/v) ABIL EN 90. To initiate the reaction, a catalyst oil phase was prepared, mixing TEMED and the oil phase at 1% (v/v)–5% (v/v). Eight‐arm PEG‐VS 20 kDa and m‐arm PEG‐ester‐SH were dissolved separately in water in the desired concentration (total PEG,% (w/v)). Furthermore, the PEG‐ester‐SH contained 0.05% (w/v) of a methacryloxyethyl thiocarbamoyl rhodamine‐B solution (10 mg in 100 µL DMSO). The exact compositions are given in the Supporting Information.

### Microfluidic Production of Rod Microgels

Rod microgels were prepared via on‐chip gelation in a flow‐focusing microfluidic device comprising a channel diameter of 80 µm employed in the plug‐flow regime based on.^[^ ^26^ ^]^ An additional flow‐focusing oil channel has been added to the channel design to ensure a homogenous flow, reducing pressure differences due to viscosity rises. To precisely control the flow rates, the microfluidic tubing (PTFE) was attached to Hamilton syringes (Gastight 1000 series, Hamilton, Reno, NV, USA) and fixed on syringe pumps (Elite 11, Harvard Apparatus, Holliston, MA, USA). The pre‐polymer solutions were filled in individual syringes, as well as the oil phase, the catalyst oil phase, and focusing oil (five syringes in total). The rod microgels were collected in the oil phase and purified subsequently by washing three times with *n*‐hexane, *iso*‐propanol, and water, respectively. The rod microgels were stored in water at 4 °C until further use.

### Degradation Experiments of Rod Microgels

Rod microgels were incubated in cell media (DMEM, Gibco) with and without enzyme at the desired concentrations inside a 96 well plate. The well plate was placed on a microscope (Leica, DFC 7000 GT) comprising CO_2_ control at 5% CO_2_ and temperature control at 37 °C. A time‐lapse was recorded to assess the degradation time of the microgels.

### Nanoindentation

Before nanoindentation, the rod microgels were immobilized on a cell culture well plate using a poly‐L‐lysine solution (0. 01% (w / v), Sigma–Aldrich). The well surface was covered with a thin film of the solution and left overnight to evaporate. The microgels were subsequently pipetted on the surface of the well plate and left for sedimentation. The mechanical properties of the rod microgels were assessed using a Pavone Nanoindenter (Optics11Life, Amsterdam, Netherlands). The Nanoindenter was equipped with a cantilever probe comprising a spherical tip radius of 9 µm and a cantilever stiffness of 0.242 N m^−1^. For each sample at least three microgels were indented. The piezo speed was set to 15 µm s^−1^ and the indentation depth to 2 µm. All measurements were conducted at r.t. and in water. The effective Young's modulus Eeff (Pa) was determined from the obtained load‐indentation curves employing the Hertz contact model. The data analysis was carried out using the Software Dataviewer V2.5 (Optics11Life).

### Swelling Behavior Microgels

For the swelling behavior, the microgels were measured in their swollen state. Then dried at room temperature and measured in their dried state. Afterward, they were resuspended in water and their reswollen state was measured after one week.

### Biofunctionalization of Rod Microgels and Sterilization

The rod microgels were post‐functionalized with GRGDSPC inside a phosphate buffer (100 mM, 94.7 mM Na_2_HPO_4_, 5.3 mM NaH_2_PO_4_, pH 8) overnight. For 1500 microgels (≈80 µm width, ≈300 µm length) 1.5 aliquots of peptide were used (25 mg mL^−1^, 10 µL/aliquot) in 300 µL buffer. Afterward, the microgels were washed five times with water. To sterilize, the microgels were incubated in ethanol (70% (v/v)) for 1 h and UV sterilized. They were again washed three times with sterile water in a sterile flow hood. Prior to cell experiments, the rod microgels were transferred into cell media by washing them three times.

### Cell Attachment Efficiency Through Live Imaging

For live imaging, sterile microgels were dispersed in methylcellulose to prevent microgel drift during imaging. 6 g of methylcellulose were sterilized and dissolved in 500 mL cell media and centrifuged at 2500 x g prior to use. The final concentration was set to 20% (v/v) of methylcellulose. The microgels and methylcellulose mixture was pipetted onto a well plate, and sedimentation of the microgels was allowed for 1 h. Subsequently, the CD10 cells were seeded on top at a concentration of 2.5 × 10^5^ mL^−1^. Every 30 s an image of the sample was taken via differential interference contrast (DIC) microscopy on a ZEISS Axio Observer Z1 inverted microscope at 37 °C and 5% CO_2_ at humid environment over a period of 120 min.

### Coating of Rod Microgels with CD10^+^ Epithelial Cells

Post‐functionalized and sterile rod microgels were dispersed in the required cell media. The CD10^+^ epithelial cells and microgels were mixed inside a LoBind Eppendorf tube at a ratio of 100 cells per microgel with a size of ≈80 µm in width and ≈300 µm in length. The total amount was calculated with respect to the number of triplicates. One triplicate contained 3000 microgels, and per well 400 µL of microgel cell dispersion was needed. The tube was then placed on an orbital shaker at 60 mpm for 1 h. The microgel cell dispersion was then pipetted onto an ultra‐low attachment 24‐well plate (Corning) and incubated for three days. Other well plates and coatings did not sufficiently inhibit the attachment of the cells (Figure S10, Supporting Information). Low attachment of the CD10^+^ cells to the well bottom was important, as the transfer into a hydrogel and complete cell coverage of the microgels was easier.

### Staining of CD10 Coated Rod Microgels

Microgel samples were washed twice for 5 min with phosphate‐buffered saline (PBS, pH 7.4, *c* = 1x, Thermo Fisher Scientific) and fixed with 4% PFA for 30 min at room temperature. After washing again for 15 min with PBS, 0.1% (v/v) TritonX‐100 (Sigma–Aldrich) was added for 3 min, and washed afterward with PBS. The samples were then incubated in 4% bovine serum albumin (BSA) for 2 h. F‐actin filaments of the cells were stained using phalloidin647 (1:1000 Abcam) in 1% BSA in PBS for 1 h, followed by washing with PBS. Cell nuclei were stained using 4′,6‐diamidino‐2‐phenylindole (DAPI) (1:200) in PBS for 20 min, followed by washing twice with PBS. ZO‐1 was stained using Invitrogen, # 40–2200 at 1:200 overnight at 4 °C and subsequently incubated with AF647 goat anti‐rabbit (1:1000 Abcam) overnight in PBS.

### Transfer Into 3D PEGQK Hydrogel Matrix

The CD10‐coated microgels were harvest from the well plate and centrifuged at 100 rcf for 5 min. Excess cell media was removed. Approximately 6000 cell‐coated microgels were diluted with 1000 µL of cell media and then used to prepare the hydrogels. The PEG hydrogel was prepared as previously described.^[^ ^29^, ^42^ ^]^ Briefly, two separate batches of eight‐arm star PEG‐vinyl sulfone (sPEG‐VS, 20 kDa; CreativePEGWorks) were conjugated with peptide solutions in triethanolamine, pH 8 (Sigma–Aldrich). The peptide sequences were H‐NQEQVSPLERCG‐NH_2_ (Q‐peptide; 1358.6 Da, GenScript, NL) and Ac‐FKGGGPQGIWGQERCG‐NH_2_ (K‐peptide; 1717.6 Da, GenScript, NL). Conjugation to cysteine residues involved Michael‐type addition by incubating the solutions for 2 h at 37 °C. The solutions were then dialyzed for 4 days against water at 4 °C to remove any unreacted peptides. The solutions were lyophilized, dissolved in water, UV sterilized, and stored at −20 °C until further use. The ^1^H NMR spectra of the compounds can be found in Figure S11 (Supporting Information). For gel preparation, equimolar amounts of the two PEG conjugates were mixed at a total concentration of 1.5% (w/v) in cell culture medium, along with a 10x calcium buffer (0.1 M CaCl_2_, 0.5 m Tris, 1.1 m NaCl (Sigma–Aldrich)), and 600 µm of the cell adhesion peptide GRGDSPC (CPC Scientific, Milpitas, CA, USA) and CD10 cell coated microgels. Gelation was initiated by adding 1250 U FXIIIa (CSL Behring, King of Prussia, PA, USA), which was activated by diluting 200 U mL^−1^ thrombin (Sigma–Aldrich) to 20 U mL^−1^ in a buffer (25 mm CaCl_2_, 10 mm Tris, and 150 mm NaCl) and incubating with the FXIII pro‐enzyme for 30 min at 37 °C, shaking gently every 5 min. The FXIIIa was then aliquoted and stored at −80 °C until further use. The hydrogel mix was pipetted in 15 µL droplets into 8‐well ibidi plates and flipped to ensure the distribution of the microgels in three dimensions. The hydrogels were flipped back after 5 min and incubated at 37 °C in a 5% CO_2_ atmosphere to complete the gelation. EGM‐2 medium (Lonza, # EBM‐2 cc‐3154, EGM‐2 singleQuots Supplements cc‐4176) with added 1% Pen/Strep was added to the hydrogels and incubated for 7 days at 37 °C in a 5% CO_2_ atmosphere. Lipase was added with a concentration of 0.000625% (w/v) at day 2 and removed at day 5.

### Transfer Into 3D PEGQK Hydrogel Matrix (Triple Co‐Culture)

The CD10^+^ coated microgels were harvested from the well plate and centrifuged at 100 rcf for 5 min. Excess cell media was removed. Approximately 6000 cell‐coated microgels were diluted with 1000 µL of cell media and then used to prepare the hydrogels. The cell concentration of the CD31^+^ and PDGFRβ^+^ cell was found to be best at a 1:1 ratio with 500 cells µL^−1^ hydrogel for each cell type. The PEG hydrogel was prepared as previously described above. The hydrogels were incubated at 37 °C in a 5% CO_2_ atmosphere to complete the gelation. EGM‐2 medium (Lonza, # EBM‐2 cc‐3154, EGM‐2 singleQuots Supplements cc‐4176) with added 1% Pen/Strep was added to the hydrogels and incubated for 14 days at 37 °C in a 5% CO_2_ atmosphere. No difference was observed in cell morphology or growth rate when CD10⁺ and PDGFRβ⁺ cells were cultured in EGM‐2 medium, which was normally used for the CD31⁺ cells (data not shown). Lipase was added with a concentration of 0.000625% (w/v) at day 2 and removed at day 5.

### Metabolic Activity Assessment Of Cells

CD10^+^, CD31^+^, and PDGFRβ^+^ were seeded in a 24‐well plate at a concentration of 2000 cells cm^−2^ and incubated for 24 h. After 24 h, a solution of 10% (v/v) of almarBlue in DMEM comprising 10% FBS and 1% AMB was prepared and added to the samples. The samples were incubated for 1 h at 37 °C and 5% CO_2_. Afterward, triplicates (50 µL) of each sample were distributed on a 96 well plate and a photometric determination (excitation 530 nm, emission 590 nm) was performed using a Tecan plate reader. Between assays, the samples were incubated in the respective cell medium.

### Nanoindentation Analysis of Microgel Containing Hydrogel

Flat disk‐shaped hydrogels with a volume of 35 µL were formed in printed pluronic rings that have a radius of 3.5 mm and a height of 1.2 mm. After cross‐linking of the hydrogels, the pluronic was removed by washing with cold PBS. The mechanical properties of the hydrogels were assessed using a Pavone Nanoindenter (Optics11Life, Amsterdam, Netherlands). The Nanoindenter was equipped with a cantilever probe comprising a spherical tip radius of 9 µm and a cantilever stiffness of 0.020 N m^−1^. For each sample at least three hydrogels were indented. The piezo speed was set to 30 µm s^−1^ and the indentation depth to 1500 µm. All measurements were conducted at r.t. and in water. The effective Young's modulus Eeff (Pa) was determined from the obtained load‐indentation curves employing the Hertz contact model. The data analysis was carried out using the Software Dataviewer V2.5 (Optics11Life).

### Injury Stimulation of Cells

The hydrogel samples were incubated with 10 ng mL^−1^ TGFβ (100‐21‐10UG, Peprotech) in culture media for a total of 48 h with a refreshing of the TGFβ after 24 h.

### Fixation and Staining (Orientation + ColI + ZO1 + SLC34A2)

Hydrogel samples were washed twice for 30 min with phosphate‐buffered saline (PBS, pH 7.4, c = 1x, Thermo Fisher Scientific) and fixed with 4% PFA for 1 h at room temperature. After washing again for 30 min with PBS, 0.1% (v/v) TritonX‐100 (Sigma–Aldrich) was added for 20 min, and washed afterward with PBS. The samples were then incubated in 4% bovine serum albumin (BSA) for 4 h. After removal of the fixative, samples were washed with PBS and incubated with primary antibody at a 1:200 dilution in PBS + 1% BSA overnight at 4 °C. After removal of the primary antibody, samples were again washed three times 30 min with PBS before adding the secondary antibody 1:200 for Col1, and 1:500 of ZO1 and SLC34A2 dilution in PBS overnight at 4 °C. To image, secondary antibody was washed away three times 30 min with PBS, and samples were covered with PBS. The following antibodies were used: Col1a1(Biozol, 1310‐01, 1:200), ARL13b (Proteintech, 17711‐1‐AP, 1:200), AF405 donkey anti‐goat (Dianova, 705‐475‐147, 1:200), AF647 donkey anti‐rabbit (Dianova, 711‐605‐152, 1:200), ZO1 (ThermoFisher Scientific, #33‐9100, 1:200), SLC34A2 (Abcam, ab122431, 1:100).

### Imaging

Imaging was performed using an Opera Phenix Plus High‐Content Screening System (Perkin Elmer, Waltham, MA, USA) with a 10x or 20x water objective and the 405, 488, 568, and 647 nm laser. Z‐stacks of ≈100 µm thickness were recorded for each sample. Fluorescent imaging was done using an ECHO Revolution microscope with a 10x objective and 488, 568, and 647 nm.

### 2D Trans‐Well Cell Experiments

Cells were seeded with a density of a total 3.0 × 10^5^ cells in a 1:1 ratio of PDGFRβ^+^:CD31^+^ on the bottom compartment of a Corning transwell system (Corning Transwell 6 well plates, CLS3450‐24EA). 1.5 × 10^5^ CD10^+^ cells were seeded on the transwell. After 1 day, 75% confluency was reached, and cells were exposed to 10 ng mL^−1^ TGFβ (100‐21‐10UG, Peprotech) in their respective culture media for a total of 48 h, with a refreshing of the TGFβ after 24 h. Cells were harvested for RNA isolation after an additional 48 h of incubation after TGFβ exposure.

### Quantitative Real‐Time Polymerase Chain Reaction (qPCR)

Samples were washed with PBS, followed by RNA extraction according to manufacturer's instructions using the InvitrogenTM PureLink TM RNA Mini‐Kit (Invitrogen 12183025, 13355364). The hydrogels were disintegrated gently by pipetting using a 1000 µL pipette tip in 300 µL lysis buffer + 1:100 β‐mercaptoethanol. RNA was stored at −80 °C until further processing. RNA was reverse transcribed using the iScript cDNA Synthesis Kit (BioRad, 1708891BUN) using 200 ng total RNA as input. qRT‐PCR was carried out with SYBR Green Mastermix in the Bio‐Rad CFX96 Real Time System with C1000 Touch Thermal Cycler. The run started with 95 °C for 3 min, then 40 cycles of 95 °C for 15 s and 60 °C for 1 min, followed by 1 cycle of 95 °C for 10 s. Samples were kept on 4 °C until further processing. GADPH was used as housekeeping gene, and data was analyzed using the ΔΔCT method. Primers used:

**Table:** 

Primer	FW	RV
GAPDH	AAGTGGTGATGGGCTTCCC	GGCAAATTCAACGGCACAGT
Collagen type 1 alpha 1 chain	TGACTGGAAGAGCGGAGAGT	GTTCGGGCTGATGTA

### Cultivation of Cell Lines

Cell lines were generated from the healthy part of human nephrectomies, immortalized, and genetically tagged as described in Bouwens et al. (2024). CD31^+^ cells were maintained in EGM‐2 medium (Lonza, # EBM‐2 cc‐3154, EGM‐2 singleQuots Supplements cc‐4176) with added 1% Pen/Strep. CD10^+^ cells were cultured in DMEM/F12 (1:1) + Glutamax (Gibco, # 31331) enriched with 10% FCS and 1% Pen/Strep. PDGFRβ^+^ were cultured in low‐glucose DMEM media (Thermo Fisher # 31885) enriched with 5% FCS and 1% Pen/Strep.

### Protein Quantification

For the hydrogel tripe co‐cultures, images were taken in 10x and 20x using Opera Phenix Plus High‐Content Screening System (Perkin Elmer, Waltham, MA, USA). Z‐stacks of ≈100 µm thickness were recorded for each sample. Using the Qupath Pixel classifier,^[^ ^43^ ^]^ a threshold was determined in the control samples above which a pixel was positive, and below which a pixel was negative for the marker. Equal areas between conditions were classified based on this threshold, and values were expressed as Positive Area in % of Collagen.

### Statistical Analysis

Data points were means ± standard errors. Statistical significance was determined by one‐way ANOVA with Tukey's multiple comparison test (^*^ *p* <0.5; ^**^ *p* <0.01; ^***^ *p* <0.001; ^****^ *p* <0.0001) *using* GraphPad Prism v10 (GraphPad Software, Boston, MA, USA).

## Conflict of Interest

The authors declare no conflict of interest.

## Supporting information



Supporting Information

## Data Availability

The data that support the findings of this study are available from the corresponding author upon reasonable request.
