# Supplementary material for: Development of a Synthetic 3D Platform for Compartmentalized Kidney In Vitro Disease Modeling
Source: Adv Healthc Mater. 2025 Oct 23;15(8):e03287. doi: 10.1002/adhm.202503287 (PMC12927530; doi:10.1002/adhm.202503287)
Supplement: Supplementary file 1 — Supporting Information [file ADHM-15-0-s001.docx]

Supporting Information

Development of a synthetic 3D platform for compartmentalized kidney *in vitro* disease modeling

Ninon Möhl^a^, Daphne Bouwens^a^, Johanna Abele, Aline Hans, Tanja Topic, Daniel Günther, Jitske Jansen, Rafael Kramann*, Laura De Laporte*

1. Microfluidic production compositions and flowrates

**Table S1.** Microfluidic prepolymer solutions compositions with respect to the used m-arm PEG-ester-SH. For all microfluidic conditions 8 arm PEG-VS 20 kDa was used and the different components were introduced stoichiometrically.

| m-arm PEG-ester-SH | Polymer concentration [w/v %] | Base concentration in O_2_ [v/v %] |
| --- | --- | --- |
| 2 arm 1.7 kDa | 15 | 4.5 |
| 4 arm 10 kDa | 5 | 1 |
| 8 arm 20 kDa | 5 | 1 |

**Table S2.** Microfluidic production settings. VS/ SH represent the flow rate for the prepolymer solutions individually. O1 is the oil phase and O2 is the oil phase containing the base initiator.

|  | Flow rate VS/SH [µL hr^-1^] | Flow rate O1 [µL hr^-1^] | Flow rate O2 [µL hr^-1^] | Flow rate Flush [µL hr^-1^] |
| --- | --- | --- | --- | --- |
| 2 arm | 30 | 55-80 | 60 | 500 |
| 4 arm |  |  |  |  |
| 8 arm |  |  |  |  |

2. Swelling behavior of microfluidic rod microgels


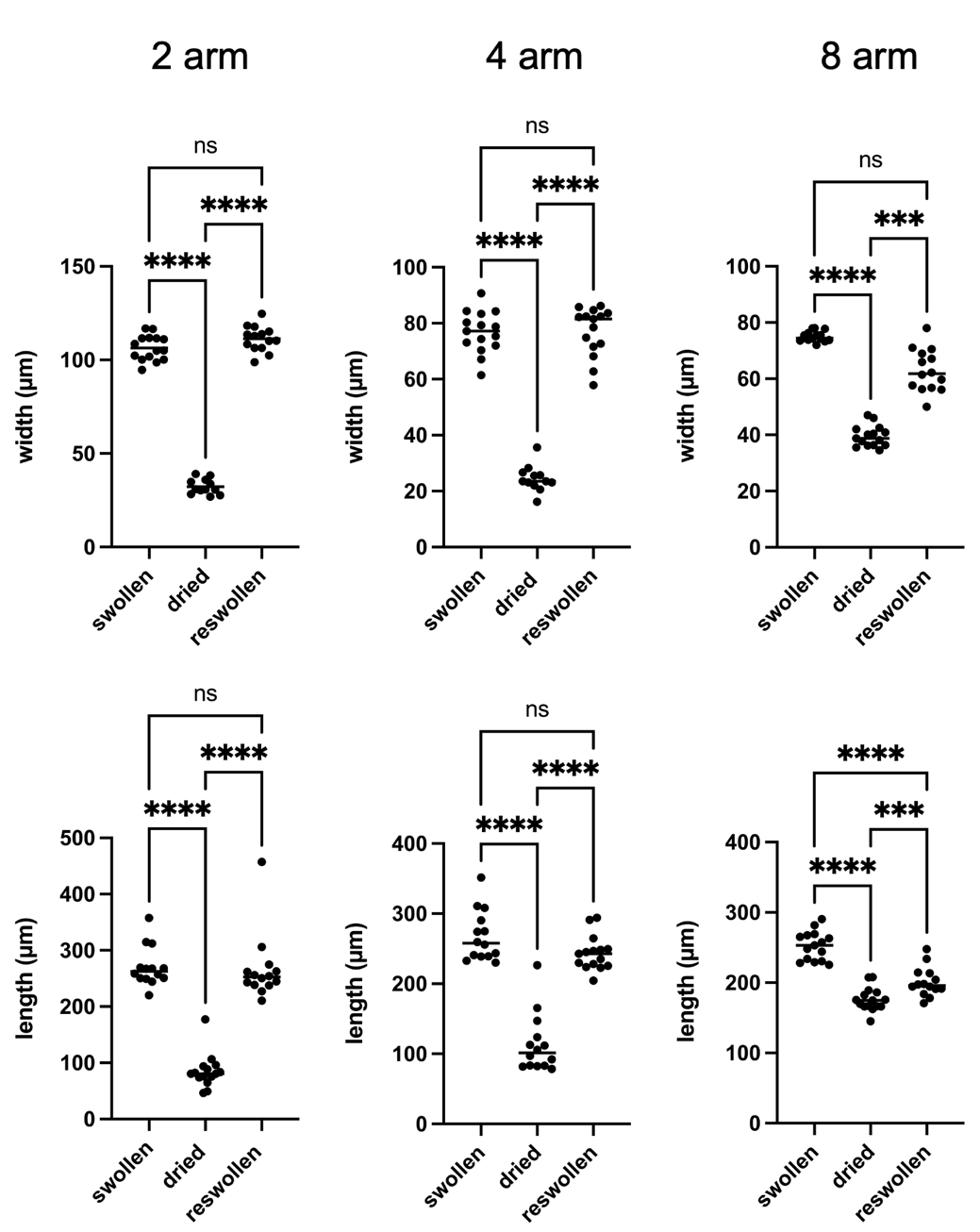
3. Metabolic assay

**Figure S1**. Swelling behavior of obtained rod microgels with different m-arm PEG-ester-SH. Data are means ± standard errors. Statistical significance was determined by one-way ANOVA with Tukey’s multiple comparison test (*p < 0.05; **p < 0.01; ***p < 0.001; ****p < 0.0001)


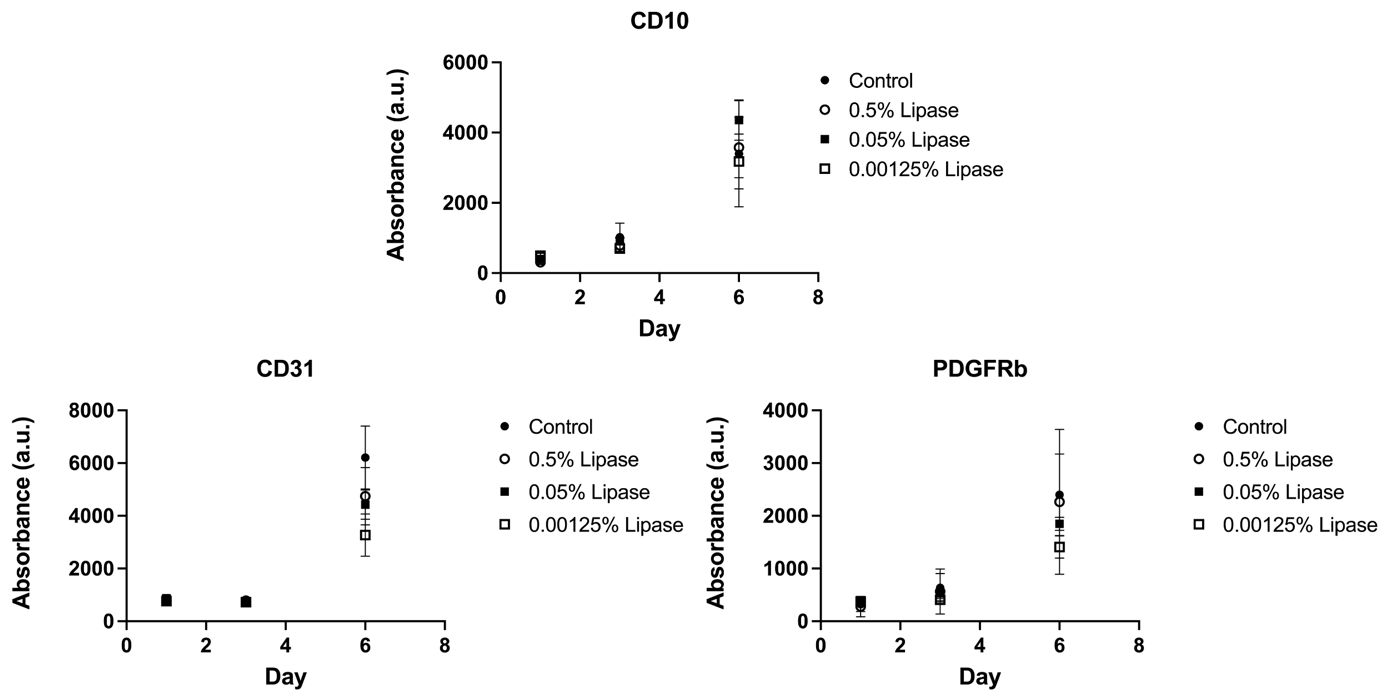


**Figure S2.** Kidney cells and lipase compatibility was assessed through a metabolic assay**.** Lipase was added from days 1-3.


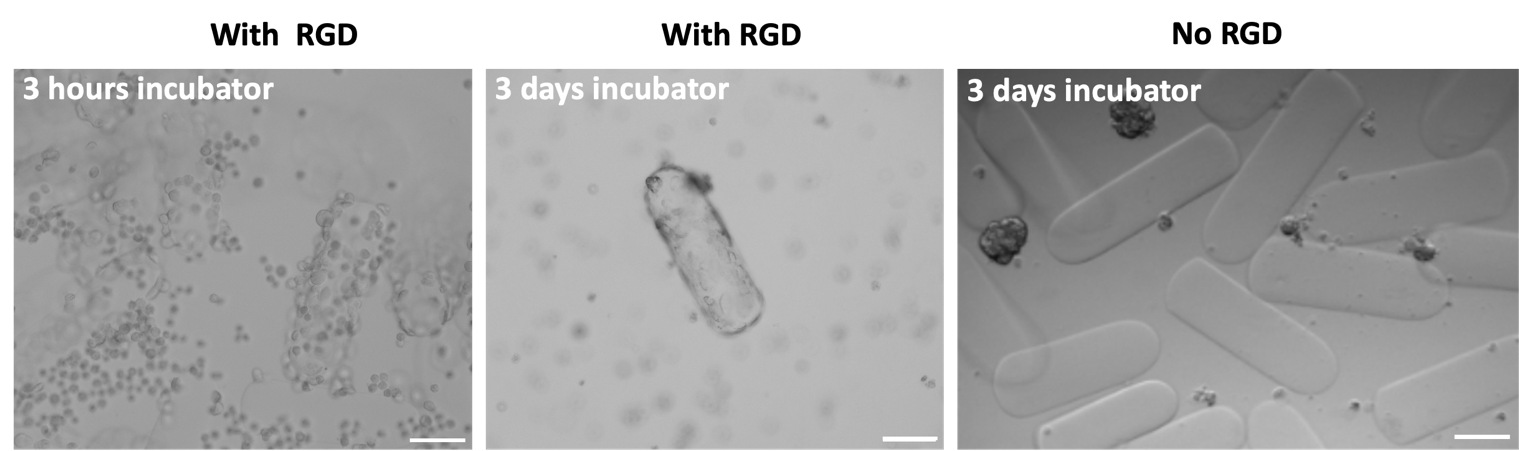
**4. CD10 epithelial cells on rod microgels with and without peptide-functionalization**

**Figure S3.** GRGDSPC-functionalized rod microgels vs unfunctionalized microgels. CD10^+^ cells were seeded at a concentration of 250 000 cells ml^-1^ with rod microgels and were cultured for three days. CD10^+^ cells do not attach to unfunctionalized rod microgels (control), proving the necessity of the cell adhesive peptide. Scale bars = 100 µm.

5. Cell attachment efficiency of CD10 epithelial cells


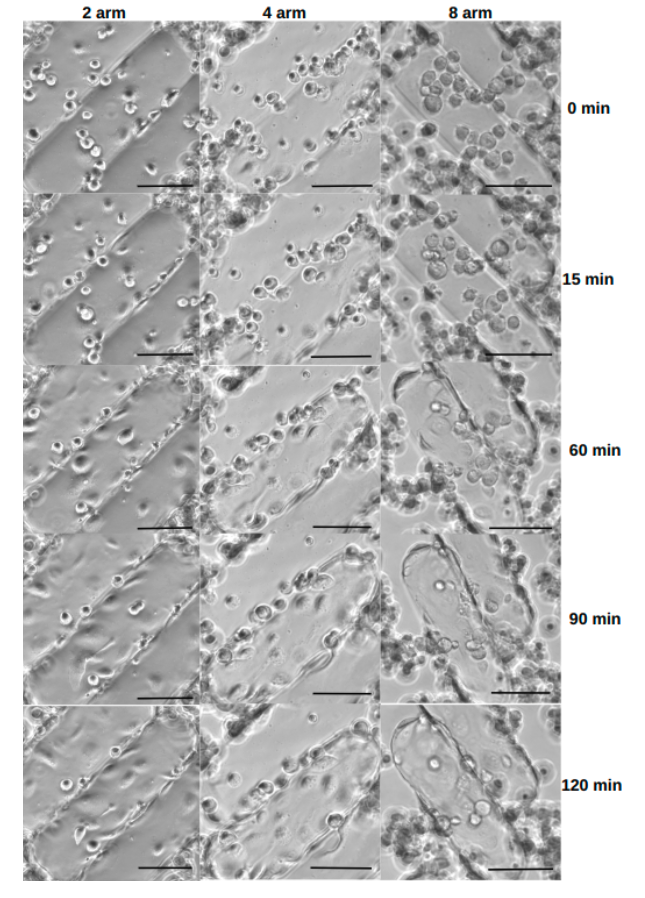


**Figure S4. Cell attachment on different microgel compositions.** Live imaging of CD10^+^ epithelial cells on GRGDSPC-functionalized microgels made of 2, 4, and 8 arm PEG-ester-SH with 8 arm PEG-VS 20 kDa. Images were recorded for a period of two hours with a concentration of 250 000 cells ml^-1^. Scale bars = 100 µm.

6. Optimization of CD31 endothelial and PDGFRb pericyte cells inside 3D PEG-QK hydrogel matrix
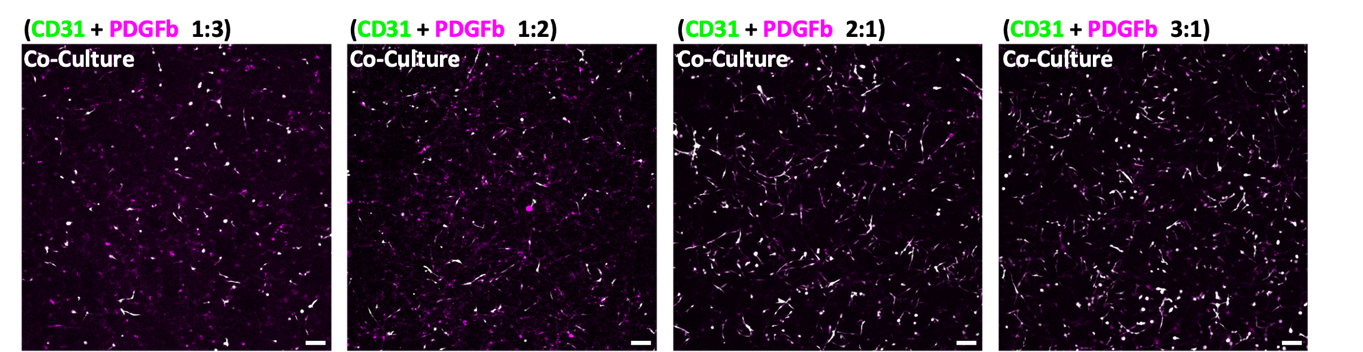


**Figure S5.** Optimization of CD31^+^ to PDGFRβ^+^ cell ratio inside a 1.5% (w/v) PEG-QK hydrogel matrix. The hydrogel contained 600 µM GRGDSPC peptide and a total of 1000 cells µL^-1^. The samples were incubated for seven days and fixed subsequently. Scale bars = 100 µm.


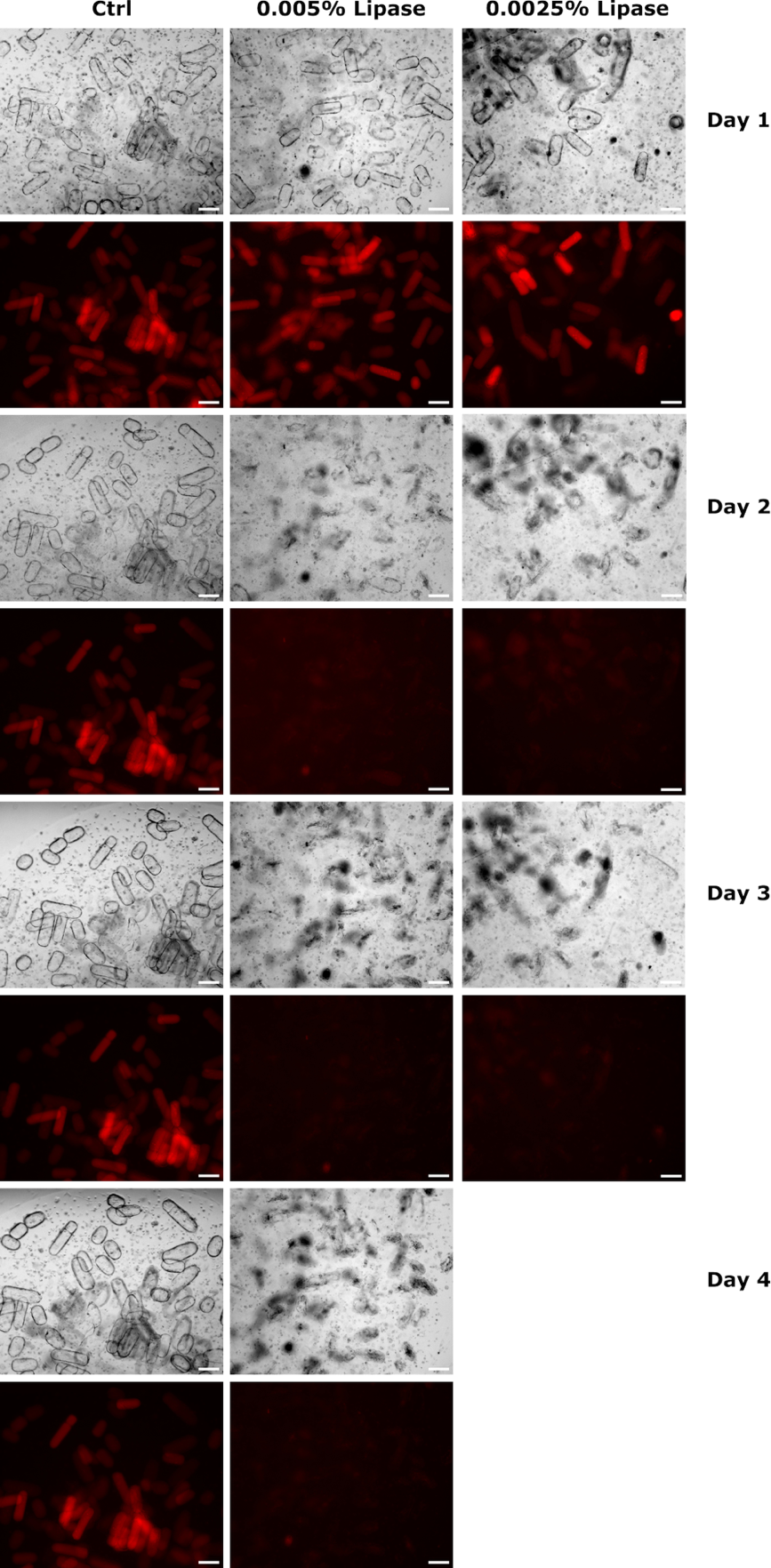
7. Degradation of rod microgels inside 3D PEG-QK hydrogel matrix

Figure S6. Degradation study inside 1.5% (w/v) PEG-QK hydrogel matrix with different lipase concentrations. Brightfield (top) and fluorescent (bottom) of Rhodamine B tagged microgels (red) to follow degradation on consecutive days inside a hydrogel matrix. The microgels are coated with CD10 epithelial cells. The hydrogel contains CD31^+^ and PDGFRβ^+^ cells in a 1:1 ratio with in total 1000 cells µL^-1^, as well as 600 µM GRGDSPC peptide. Scale bars = 200 µm.

8. Different PEGQK concentrations at different time points


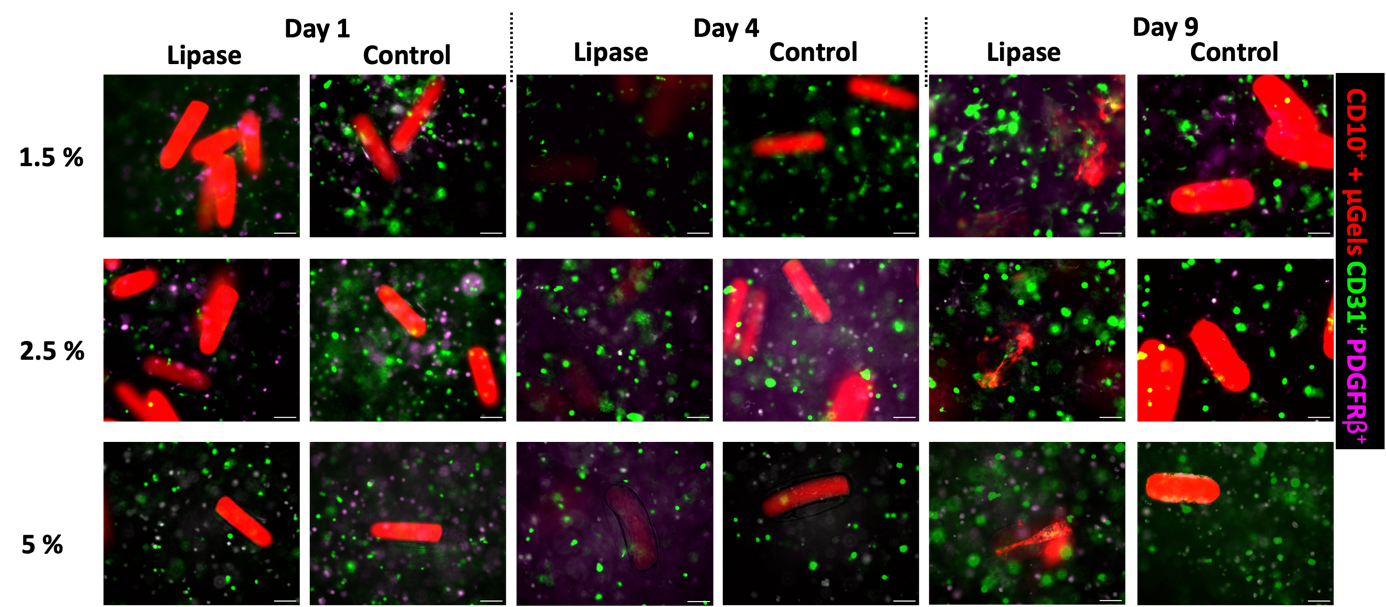


**Figure S7.** Fluorescent images of 3D compartmentalized triple-co-culture system with different PEG-QK polymer concentrations (1.5, 2.5 and 5% (w/v)) at different time points. Lipase (0.000625% (w/v)) was added on day two and removed on day five to degrade the rod microgels. Scale bars = 100 µm.


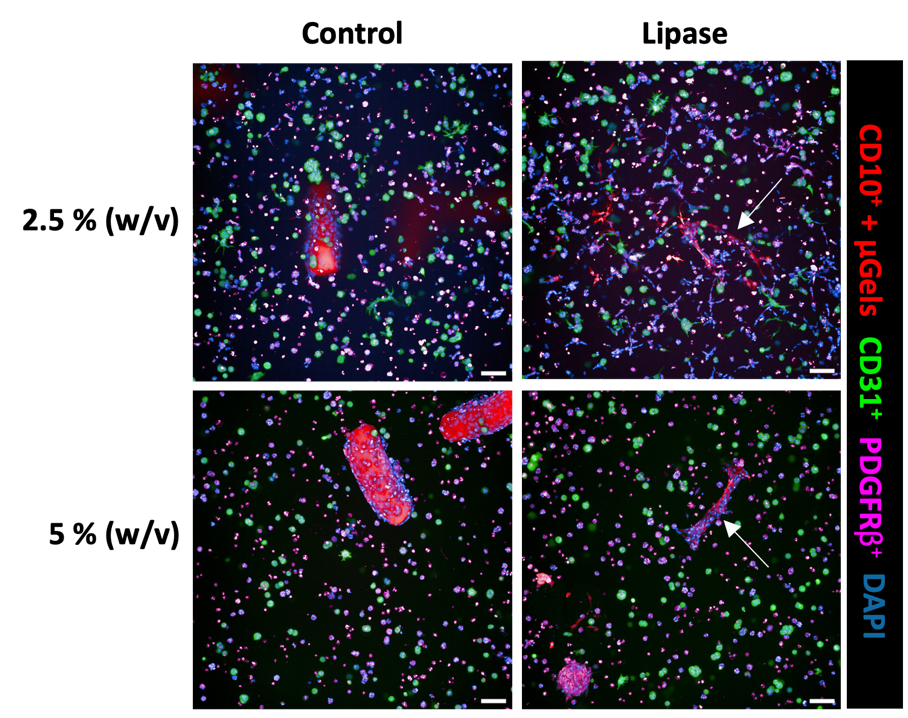
9. Different PEGQK concentrations after 14d

**Figure S8.** Triple co-culture with higher PEG-QK concentrations after 14 days. Confocal images after microgel degradation show shrinkage of the CD10^+^ layer (indicated by white arrow) and do not keep the tubular shape in the stiffer hydrogels. Furthermore, there is no sufficient network formation. Scale bars= 100 µm.

10. Col1 protein expression in the transwell system


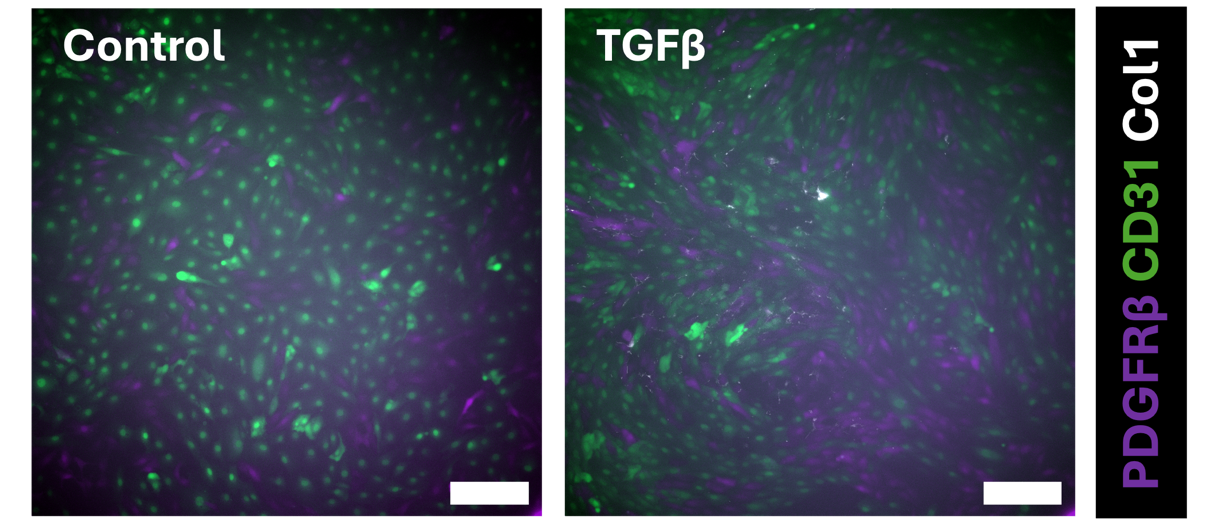


Figure S9. Collagen1 protein expression in CD31^+^ and PDGFRβ^+^ coculture. A triple co-culture in the transwell system consisted of a separated CD10^+^ cell compartment, and CD31^+^ and PDGFRβ^+^ cells seeded on the bottom of a 6-well plate. Representative IF images reveal a slight upregulation in Col1 in the TGFβ treated cells, but not in the same magnitude as in the hydrogels. Scale bars = 200 µm.

11. Different well plate types and coating for CD10 on microgel culture


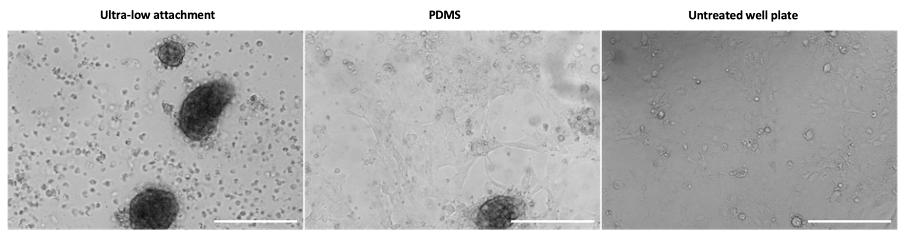


**Figure S10.** Different surface coatings to prevent CD10^+^ cell attachment. Cells cultured for three days in an ultra-low attachment well plate (Corning costar) did not adhere to the well bottom. On the other hand, cell culture well plates (labsolute) treated with a PDMS surface coating still show cell attachment, though not as much as the control (untreated well plate). Scale bars = 250 µm.

**11. Characterization of PEG-QK compounds using NMR**


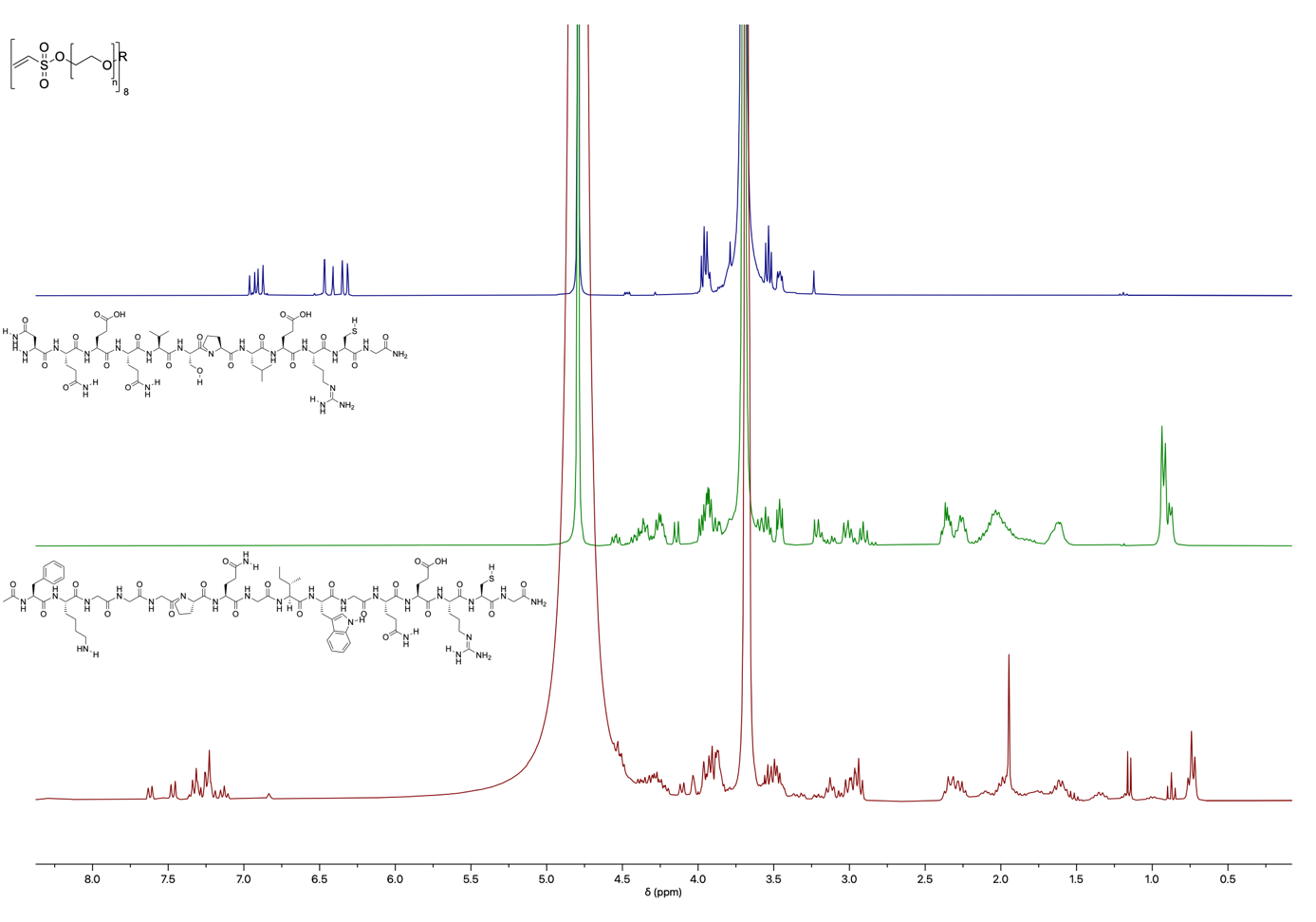


**Figure S11.** ^1^H NMR of PEG-VS (top), PEG-Q (middle), and PEG-K (bottom). The compounds were synthesized according to a previous publication.^[1]^

For PEG-K the aromatic peaks are visible from 7.1 ppm to 7.7 ppm. For PEG-Q the isobutyl group can be found at 0.82 ppm. The PEG-VS signals vanish completely upon addition of the peptides.

1H NMR (D20, 300 MHz): PEG-K: δ 7.54 (d, *J* = 7.8 Hz, 1H), 7.38 (d, *J* = 8.1 Hz, 1H), 7.31 – 6.99 (m, 8H), 3.60 (m, 227H). PEG-Q: δ 3.60 (m, 227H), 0.81 (m, 12H). PEG-VS: δ 6.89 (dd, 1H), 6.42 (d, 1H), 6.31 (d, 1H), 3.68 (m, 227H).

1. Licht, C., et al., *Synthetic 3D PEG-Anisogel Tailored with Fibronectin Fragments Induce Aligned Nerve Extension.* Biomacromolecules, 2019. **20**(11): p. 4075-4087.
